# Asthma diagnosis and treatment – 1013. Six month follow up on pediatric patients with severe persistent allergic asthma after on eyear of omalizumb therapy

**DOI:** 10.1186/1939-4551-6-S1-P13

**Published:** 2013-04-23

**Authors:** Tarun Choudhuri

**Affiliations:** 1 Pediatrics, Fiap, Kolkata, India

## Purpose

Anti-IgE antibody had been used in severe persistent allergic asthma in adults. However, its long-term efficacy in pediatric patients in India has not been reported.

## Method

20 pediatric (12 male and 8 female) patients, with mean age of 8.5 having severe persistent allergic asthma, with recurrent exacerbations and on oral/IV steroids, prospectively received Omalizumab 150mg/300mg, depending on IgE & body weight for 6 month to 1 year,at B.R.Singh Hospital, Kolkata. Number of exacerbations, total dose of oral Steroids, use of rescue medications, ICS/LABA dose used were recorded at the baseline, 24 weeks, end of treatment and on 6 month follow up (between February 2011 and August 2012) and statistically analyzed.

## Results

All 20 patients after anti-IgE therapy were followed-up for 6months.Significant reductions observed in total oral steroid use by six months -15.5mg (27.5mg vs. 12mg; 95%CI, *p*<*0.001*). Use of rescue medications with β2 agonists inhalerdecreased by -7.90 puffs (12.0 vs 4.10 puffs; 95%CI, p-value <0.001) at 24 weeks and by -13.67 puffs (13.67 vs 0.00 puffs; 95%CI, p-value <0.001) at 52 weeks (n=6). There was significant reduction in ICS dose at week 24 by -287.50 mcg (550.0 vs 262.50 mcg; 95%CI, p-value <0.001). By week 52 (n=6) ICS dose further declined to -458.33 mcg (750.0 vs 291.67 mcg; 95%CI, p-value <0.001). 15 (75%) patients had severe asthma symptoms at baseline,which came down to 6 (30%) (*p*<*0.05*), at week 24. Similarly 15 (75%) patients had unscheduled hospitalization at baseline, this figure came down to zero by week 24.

## Conclusion

Use of anti-IgE antibody for 6 months to 1 year is well tolerated and led to overall significant improvement and stabilization of pediatric patients with severe persistent allergic asthma which was maintained at 6 months post treatment follow-up period.

